# Trajectories of vision in older people: the role of age and social position

**DOI:** 10.1007/s10433-015-0360-1

**Published:** 2016-01-04

**Authors:** Jennifer Whillans, James Nazroo, Katey Matthews

**Affiliations:** 1 The Cathie Marsh Institute for Social Research (CMIST), School of Social Sciences, University of Manchester, Humanities Bridgeford Street-G18, Manchester, M13 9PL UK; 2 The Cathie Marsh Institute for Social Research (CMIST), School of Social Sciences, University of Manchester, Humanities Bridgeford Street-G29, Manchester, M13 9PL UK; 3 The Cathie Marsh Institute for Social Research (CMIST), School of Social Sciences, University of Manchester, Humanities Bridgeford Street-G21, Manchester, M13 9PL UK

**Keywords:** Self-reported vision, Visual function, Health inequality, Longitudinal study, Optimal matching, Sequence analysis

## Abstract

Visual impairment becomes more prevalent with age. Rather than a uniform decline in vision with age, the strength and direction of change varies between people. This study applies an analytical method that posits multiple trajectories in eyesight, allowing for a more specific description of developmental course of this health outcome and its relationship with social position. The analysis uses the responses of 2956 respondents, aged 60 years and over, followed over 8 years (five observations) as part of the English longitudinal study of ageing. At each observation respondents self-reported their general vision. Optimal matching (sequence analysis), hierarchical clustering, and multinomial logistic regression were used to describe the sequential data, produce a typology of vision trajectories, and examine the socio-demographic characteristics associated with different trajectories. Eight distinctive clusters of trajectories were identified. The probability of reporting different types of vision trajectory varies with a change in age; however, the magnitude of the age effect is associated with social position. Visual impairment in older people is an increasingly relevant area for policy focus, with the rapid growth and diversity of the older population. Identifying factors underpinning the patterning of changes in visual function is essential for developing evidence-based policy, which both meets the needs of those most at risk and increases cost-effectiveness of public health interventions.

## Introduction

Vision impairment in older people is an increasingly relevant area for public policy initiative. Increasing life expectancy will result in increasing numbers of older and dependent people who will depend on state-provided health and social services (Marmot and Nazroo 2001). Additionally, the older population is diverse, with marked socioeconomic differences in morbidity and likely differences in the impact of illness according to an older individual’s social circumstances (McMunn et al. 2009) so identifying and addressing social inequalities in vision will be of increasing concern for public policy (Marmot and Nazroo 2001). The aim of this paper is to provide evidence of the different trajectories of self-reported vision in older people. The typology will be examined to identify the socio-demographic factors associated with different vision trajectories, thus describing social inequalities in changes in vision.

While cross-sectional studies have shown trends in trajectories of vision by comparing the vision of different age groups, longitudinal studies can instead establish a sequence of observations on the same individuals. Information is gathered on individuals’ trajectories on a given measure, enabling analyses of between-person differences in within-person change. Compared with approaches that measure the subjects’ vision at only on one or two time points, longitudinal data offers an understanding of the developmental course, causes, and consequences of the individuals’ changes in vision.

Longitudinal approaches often rely on normative trajectory approaches, which summarize the observations across multiple measurement occasions, giving a trajectory. Normative trajectory approaches can readily incorporate measures of stable factors (e.g., gender) and time-varying factors (e.g., diagnosed illness) that may be associated with patterns of vision change (Maggs and Schulenberg 2004). However, more specific trajectories of vision change are likely to be concealed within the normative trajectory of vision change; that is, rather than a uniform decline in visual function with age, it is likely that the strength and direction of change varies between people. Some trajectories are likely to be more straightforward, such as stable good or poor vision; other trajectories may be more complex with fluctuation between low and high levels of vision, perhaps related to medical intervention. A single averaged trajectory could mask important differences, overlooking the multidimensional nature of both social inequalities and of health itself (Singh-Manoux et al. 2003). A multiple-trajectory approach identifies distinct subgroups of people who follow similar trajectories over time, allowing for more specific understanding of the origins and developmental course of health outcomes (Maggs and Schulenberg 2004).

## Methods

### Sample

The English Longitudinal Study of Ageing (ELSA) contains detailed information on the health, economic, and social circumstances of older people in England. At the time of writing, five waves of data are currently available: the first was conducted in 2002/2003 and the survey is repeated every 2 years, with the final wave conducted in 2010/2011. The core sample for wave 1 was drawn from households that had previously responded to the Health Survey for England (HSE) in 1998, 1999, or 2001. Respondents were selected if they were aged 50 and over at the beginning of March 2002. The partners of the core members were also interviewed and in waves three and four refreshment samples were selected from HSE 2001–2004 and 2006.

The sample used for the present analysis was restricted by three criteria. First, the sample was limited to core members who responded to the first five waves of ELSA, reducing the sample from 11,391 to 5262. For this subsample, a longitudinal weight was available that adjusts for the non-response to HSE and each of the four previous waves of ELSA. Second, six respondents were excluded who had not answered the question on self-reported general vision in all five waves. These first two criteria were necessary as the analysis of change was based on a full sequence of data, rather than simply examining differences between the first and last observations. Finally, respondents aged under 60 at wave 1 were excluded because preliminary analysis indicated that the most notable changes in vision trajectories occurred in the over 60 s population. So, the analysis used the sequence of responses of 2956 respondents, aged 60 and over, who self-reported general vision in five successive waves of ELSA (Table 1).

**Table 1:** Sample characteristics

	*N*	Unweighted (%)	Weighted (%)
Total *N*	2956		
Gender			
Men	1261	42.7	42.9
Women	1695	57.3	57.1
Ethnicity			
White	2909	98.4	97.4
Non-white	47	1.6	2.6
Age at wave 1			
Men			
60–64	388	30.8	31.8
65–69	375	29.7	28.2
70–74	274	21.7	20.8
75–79	153	12.1	13.1
80+	71	5.6	6.2
Women			
60–64	492	29.0	27.2
65–69	488	28.8	25.2
70–74	349	20.6	21.0
75–79	223	13.2	15.6
80+	144	8.5	10.9
Wealth quintile			
Highest	407	13.9	17.6
Fourth	549	18.8	19.5
Middle	606	20.7	20.4
Second	626	21.4	20.3
Lowest	734	25.1	22.2
SSS quintile			
Highest	92	3.2	3.2
Fourth	453	15.9	15.9
Middle	1318	46.2	46.2
Second	854	29.9	29.9
Lowest	139	4.9	4.9
Executive function			
Optimal	1804	65.39	61.31
Suboptimal	1138	34.61	38.69
Memory function			
Optimal	1826	61.31	62.05
Suboptimal	1116	38.69	37.95
Treatment and eye diagnoses			
Cataract surgery	657	22.2	23.4
Glaucoma	337	11.4	11.9
Diabetic retinopathy	106	3.6	3.8
AMD	270	9.1	9.1
Cataracts	1404	47.5	48.1

Attrition due to the death and decline in the health of participants can cause particular problems in cohort studies of older people. Analyses of data from participants who continue in a study over many waves are potentially biased towards those who are healthy enough to do so (Brilleman et al. 2010). Table 2 shows cross tabulations of those aged 60 and over included and excluded from the final sample by self-rated vision at wave 1 and socio-demographic factors at wave 1. These descriptive statistics suggest people with poorer vision are less likely to be included in the final sample, as are those who are older, in poorer wealth quintiles, in lower self-perceived social status quintiles and who have an eye disease. The ELSA data provides a set of longitudinal weights in order to account for potential bias arising from attrition, and these will be applied to all analyses in this instance.

**Table 2:** Characteristics of participants included and excluded from sample (weighted, excluded participants are inclusive only of those aged 60 and over)

	Included	Excluded	*χ* ^2^ (df) and *p* value of *χ* ^2^ (included vs. excluded)
	(*N* = 2956) (%)	(*N* = 4179) (%)	
Vision at wave 1			120.79 (4df) *p* = 0.000
Excellent or v. good	47.32	38.86	
Good	39.34	38.46	
Fair	10.51	15.55	
Poor or blind	2.82	6.99	
Missing	0.00	0.14	
Gender			8.27 (1df) *p* = 0.004
Men	42.79	46.28	
Women	57.21	53.72	
Ethnicity			21.97 (1df) *p* = 0.000
White	98.42	96.62	
Non-white	1.58	3.38	
Age at wave 1			440.23 (4df) *p* = 0.000
60–64	30.75	19.54	
65–69	27.36	18.61	
70–74	20.63	19.51	
75–79	14.00	18.42	
80+	7.25	23.91	
Wealth quintile at wave 1			294.68 (5df) *p* = 0.000
Highest (wealthiest)	24.56	15.07	
Fourth	21.11	16.98	
Middle	20.59	19.46	
Second	18.42	20.99	
Lowest (poorest)	14.14	27.50	
Missing	1.17	0.00	
SSS at wave 1			80.48 (5df) *p* = 0.000
Highest (wealthiest)	4.75	3.65	
Fourth	28.53	22.30	
Middle	44.49	43.27	
Second	15.49	20.63	
Lowest (poorest)	3.13	5.07	
Missing	3.62	5.09	
Treatment and eye diagnoses at wave 1			
Cataract surgery	7.15	12.11	44.29 (1df) *p* = 0.000
Glaucoma	5.36	5.97	1.54 (1df) *p* = 0.215
Diabetic retinopathy	1.21	2.13	7.03 (1df) *p* = 0.008
AMD	1.71	2.70	6.50 (1df) *p* = 0.011
Cataracts	16.02	23.15	51.56 (1df) *p* = 0.000

### Visual function measures

As ELSA does not collect clinical measures of visual acuity, a self-reported measure of general vision is used instead. Self-reported vision is captured using the question *Is your eyesight (using glasses or corrective lenses as usual)* *excellent, very good, good, fair, or poor?* An additional response, *registered* *blind*, was included where respondents spontaneously provided this answer. For ease of model interpretation, the responses *excellent* and *very good* were amalgamated to create a larger group of individuals with optimal vision. Additionally, *poor* and *blind* were also combined, as the number of people reporting blindness at each wave was very small (10, 12, 10, 26, and 33 respondents at waves 1–5, respectively). A discussion of how self-reported measures of vision compare to acuity is provided in the final section of this paper.

### Other measures included in the analysis

#### Age

Age groups were entered into the model to control for the non-linear effect of age. Respondents were grouped into 5-year bands according to age at wave 1 (Age 60–64, 65–69, 70–74, 75–79, and 80 plus years). For interpretation it should be noted that those in the cohort ‘60–64 years at wave 1’ were aged 68–72 in wave 5. The youngest cohort was used as the reference category.

#### Wealth

Wealth is measured in quintiles of net total non-pension wealth measured at benefit unit (household) level at wave 1. This includes, for example, the value of the primary house minus the outstanding primary house mortgage, the value of savings and shares minus credit card debts and loans, and the value of other properties and businesses. Wealth is argued to reflect command over material resources better than any other measure of socioeconomic status and, unlike education and occupational class, it reflects older peoples’ life-time cumulative social status (de Oliveira et al. 2010; Demakakos et al. 2008).

#### Subjective social status

Respondents’ perception of their social positions is also found to be a significant predictor of health (Cundiff et al. 2011; Demakakos et al. 2008). Subjective social status (SSS) was measured using a scale represented by a 10-rung ladder. Respondents are asked to identify their social standing in society (somewhere between best and worst off) on the rung on which they feel they stand. In a study using ELSA data, Demakakos et al. (2008) found that SSS partially or fully mediated the associations between education, occupational class, and wealth when the outcome was a self-reported health measure. Given evidence of their independent effects on health measures, both wealth and SSS were entered into the model, cumulatively controlling for the effects of social position. Again, SSS responses were categorized into quintiles. For both wealth and SSS, the highest quintiles were used as reference categories.

#### Cognitive function

Cognitive decline is associated with both visual impairment in older age (Wahl and Heyl 2003; Lin et al. 2004) and social position (Cagney and Lauderdale 2002). These analyses use memory and executive function indices to control for cognitive ability. The memory function index asks respondents to identify the day and date (time orientation) and to participate in a word recall task, originally developed for the Established Populations for Epidemiological Study of the Elderly in Iowa (O’Hara et al. 1986) and a prospective memory task, based on a task used in the Medical Research Council Cognitive Function and Ageing Study (MRC CFAS Study 1998), and has a score range of 0–29. The executive function index is comprised of a letter cancellation task, originally developed for the 1946 birth cohort study (Richards et al. 1999), and a verbal fluency task, where respondents are asked to name as many animals as possible in 1 min, and has a score range of 0–20. Both indices are included in models as binary variables, showing optimal function (upper two tertiles of function) compared to suboptimal function (the lowest tertile of function).

#### Eye disease

ELSA contains information on diagnosed eye diseases and medical treatment. The most common causes of vision loss and blindness in older people are glaucoma, diabetic retinopathy, age-related macular degeneration, and cataracts. As the onset of eye disease can have a severe impact on vision trajectories, the diagnosis of each of these conditions during the 8-year observation window are entered as binary variables. Cataract surgery can significantly improve vision and has both immediate effect and lasting, longer term improvements (Lundqvist and Mönestam 2006, p. 1950). The presence of cataract surgery during the 8-year observation window is entered in the model as a binary independent variable. ELSA does not contain any other information on medical or surgical vision treatments.

### Analysis

The TraMiner and Cluster packages in R were used to run optimal matching (OM), cluster analysis and to produce a typology of vision trajectories. Stata v.12.1 was then used to run multinomial logistic regression modelling (MNLM) to examine the socio-demographic characteristics associated with different trajectories. Wave 5 longitudinal weights were applied in both OM and MNLM analyses, to deal with survey non-response.

OM is used to identify patterns in sequence data without making prior assumptions about the kinds of patterns the data may contain, or the processes causing variation in Trajectories (Abbott and Tsay 2000). OM examines entire sequences rather than transitions into a particular state. All sequences consisted of five observations and each measurement of visual function was coded as being one of four states: (1) excellent or very good, (2) good, (3) fair, and (4) poor or blind. In OM, the degree of dissimilarity between trajectories is determined by ‘the least number of weighted edit operations that are necessary to turn one sequence into the other (i.e., to match the two sequences)’ (Lesnard 2010, p. 391). Dynamic hamming distance (DHD) is an advanced method of OM that uses only substitution operations, with time-varying costs inversely proportional to observed transition frequencies. The output from OM is a dissimilarity matrix for each observed sequence, which must then be combined with a data reduction procedure.

Agglomerative hierarchical clustering was applied to the resulting OM dissimilarity matrix using the Ward clustering method, to organize the sequences into meaningful groups in a way that maximizes the similarity of cases within each cluster while maximizing the dissimilarity between groups. This clustering algorithm finds successive clusters using previously established clusters; it starts with each sequence being its own cluster and successively merges them into larger clusters. Deciding how many clusters are necessary to give a synthetic, but faithful, representation of the data is a central problem in sequence analysis (Abbott and Tsay 2000; Lesnard 2010); consequently, two procedures were applied here. Aisenbrey and Fasang (2007) state the mean within cluster distance should be at least half of the mean between cluster distance, to indicate truly distinct groups (p. 15). Following this, the graphical representations of various cluster solution were examined to ensure the results were interpretable and meaningful (Abbott and Tsay 2000).

As cluster membership is a categorical variable, the resulting cluster solution was submitted as the dependent variable in multinomial logistic regression with age, sex, ethnicity, wealth, subjective social status, cognitive function, diagnosed eye diseases, and having had cataract surgery entered as independent variables. The output from MNLM was transformed into predicted probabilities, using the *prchange* command in Stata, in order to ease interpretation of the model.

## Results

### Typical vision trajectories

**Fig. 1:**
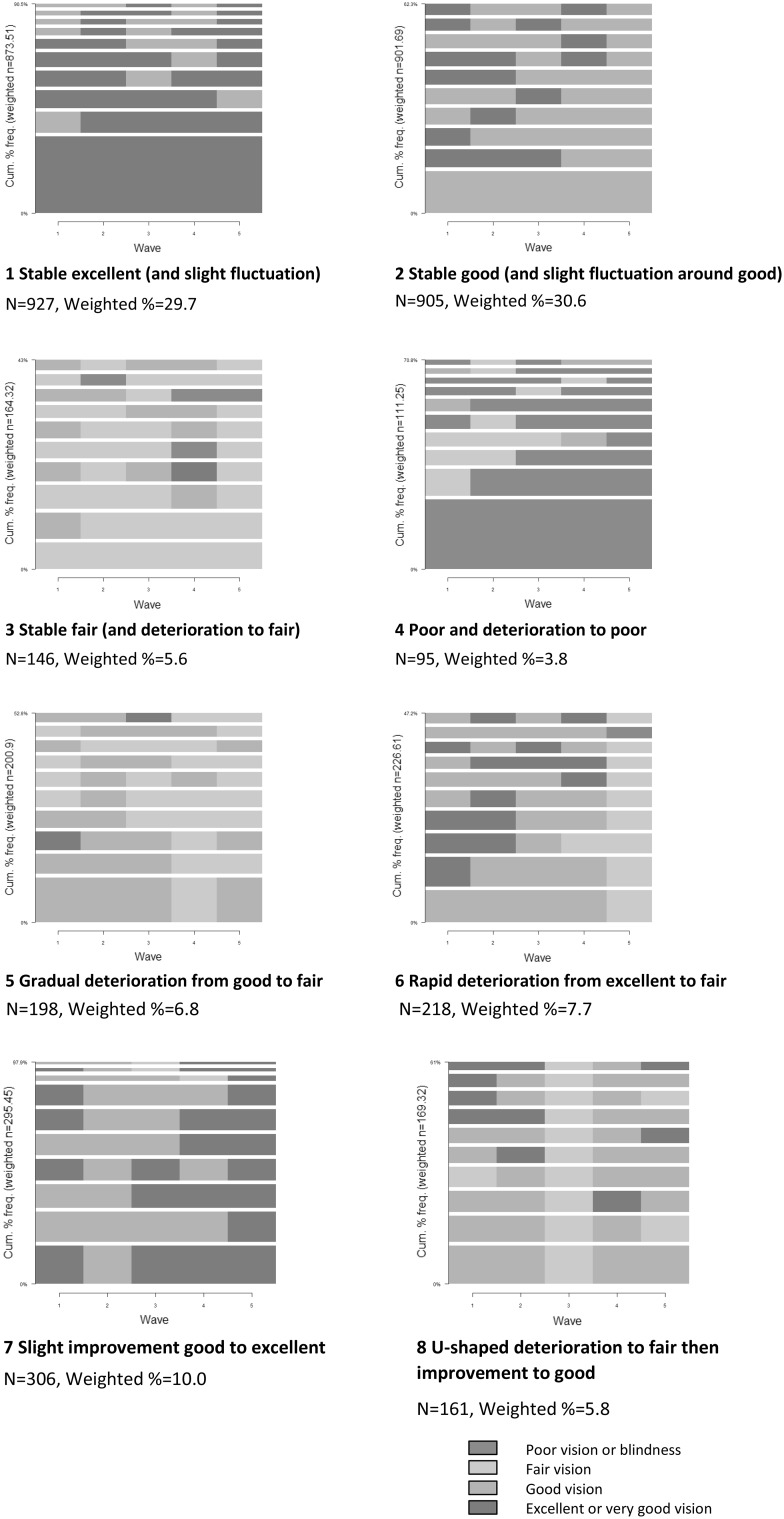
Sequence frequency plots for an 8-cluster solution. **1** Stable excellent (and slight fluctuation). *N* = 927, Weighted % = 29.7. **2** Stable good (and slight fluctuation around good). *N* = 905, Weighted % = 30.6. **3** Stable fair (and deterioration to fair). *N* = 146, Weighted % = 5.6. **4** Poor and deterioration to poor. *N* = 95, Weighted % = 3.8. **5** Gradual deterioration from good to fair. *N* = 198, Weighted % = 6.8. **6** Rapid deterioration from excellent to fair. *N* = 218, Weighted % = 7.7. **7** Slight improvement good to excellent. *N* = 306, Weighted % = 10.0. **8** U-shaped deterioration to fair then improvement to good. *N* = 161, Weighted % = 5.8

Having conducted OM, cluster analysis was used to identify groupings of similar trajectories. Aisenbrey and Fasang (2007) state a minimum of an 8-cluster solution must be used in order for the ratio of mean within cluster distance to mean between cluster distance to fall below the threshold of 0.5. Examining the sequence frequency plots for various cluster solutions showed a distinctive trajectory would have been created at a 10-cluster solution, but this produced two clusters that were somewhat superfluous. Consequently, this process indicated an 8-cluster solution suitably represented the sequences in the data. The 8-cluster solution showed trajectories reflecting stability (clusters 1–3), deterioration (clusters 4–6), and an improvement in vision (clusters 7 and 8). Table 3 shows key information on each cluster and Fig. 1 shows the set of sequence frequency plots for the 8-cluster solution, displaying the most frequent sequences in each cluster; sequences are displayed bottom-up in decreasing order of their frequencies.

**Table 3:** Characteristics of the clusters identified by the 8-cluster solution

Cluster number	Cluster description	*N*	Weighted (%)
1	Stable excellent (and slight fluctuation around excellent)	927	29.7
2	Stable good (and slight fluctuation around good)	905	30.6
3	Stable fair (and slight deterioration to fair)	146	5.6
4	Poor and deterioration to poor	95	3.8
5	Gradual deterioration from good to fair	198	6.8
6	Rapid deterioration from excellent to fair	218	7.7
7	Slight improvement from good to excellent	306	10.0
8	U-shaped deterioration to fair then improvement to good	161	5.8

Table 3 shows cluster 1, *Stable excellent vision*, is the second largest of the 8 clusters (29.7 % of all sequences analysed). Descriptive statistics (not shown) indicated at wave 1, 73.0 % of cluster 1 members self-reported excellent or very good vision; by wave 5, this increased to 89.4 %. 39.0 % of cluster 1 members reported stable excellent or very good vision across all 5 waves (*N* = 376). Cluster 2, *Stable good vision*, is the largest cluster of the 8-cluster solution (30.6 %). At wave 1, large proportions of cluster members reported good vision (50.5 %) and excellent or very good vision (38.7 %). By wave 5, almost all cluster members reported good vision (94.4 %). Clusters 1 and 2, together, account for just over 60 % of all sequences.

Clusters 3–8 categorizes the remaining 40 % of sequences and reflect trajectories containing lower levels of visual function and/or notable change in self-reported vision over time. Cluster 3, *Stable fair vision,* contains 11 observations of stable fair vision throughout the period, with the remaining 135 trajectories within this cluster showing a fluctuation around and deterioration to fair vision. In wave 1, 32.2 % of observations from cluster 3 members were self-reported fair vision and 37.9 % were good vision; by wave 5, 74.8 % of responses were self-reported fair vision. 52.8 % of all responses from cluster 3 members were self-reported fair vision.

The three clusters that describe deterioration trajectories contain 18.3 % of all sequences analysed. Cluster 4, *Poor vision and deterioration to poor,* is the smallest but a very distinctive category (*N* = 95, 3.8 %). At wave 1, just over half of the cluster reported poor vision or blindness (51.1 %), and over a third reported fair vision (38.0 %). By wave 5, 85.0 % of the cluster reported poor vision or blindness. Over a quarter of cluster 4 reported poor vision or blindness in all 5 waves (27.7 %, *N* = 26). Cluster 5 describes trajectories of *gradual deterioration from good to fair*. The most common response at wave 1 was self-reported good vision (43.9 %) and an almost equal proportion reported fair vision (40.5 %). By wave 4, 88.3 % reported fair vision although by wave 5, 42.4 % reported fair vision. Cluster 6 contains sequences showing *rapid deterioration from excellent to fair vision*. At wave 1, two fifths of the cluster reported excellent or very good vision (41.3 %) and over half reported good vision (49.1 %). By wave 5, 65.3 % reported fair vision and 34.3 % reported poor vision or blindness.

Finally, there were two clusters representing trajectories of improvement. Cluster 7, *Improvement good to excellent*, accounted for 10.1 % of sequences. At wave 1, 44.8 % reported good vision and 55.2 % reported excellent or very good vision; by wave 5, almost all respondents self-reported excellent or very good vision (99.2 %). A small number of trajectories were clustered together in cluster 8 which reflect a u-shaped trajectory of *deterioration to fair then improvement to good*. At wave 1, over half of cluster 8 reported good vision (57.1 %) and just over a quarter reported excellent or very good vision (26.0 %). At wave 3, almost all respondents reported fair vision (98.8 %). By wave 5, again over half of cluster members reported good vision (55.3 %) and a just over a fifth reported excellent or very good vision (21.6 %).

### Socio-demographic characteristics associated with cluster membership

Multinomial logistic regression modelling was used to examine how cluster membership was related to a set of socio-demographic characteristics. A combination of Pseudo *R* ^2^ and likelihood ratio tests comparing nested models were used to assess the contribution of each set of explanatory variables (Table 4). Pseudo *R* ^2^ indicated that the explanatory power of a model containing SSS (and not wealth) was greater than a model containing wealth (and not SSS), albeit that both *R* ^2^ values were low (*R* ^2^ = 0.042 and *R* ^2^ = 0.040, respectively). The inclusion of both wealth and SSS increases the explanatory power of the model beyond that of a model containing only a single measure of social position (*R* ^2^ = 0.051, LR *χ* ^2^(35) = 94.16, *p* < 0.000). The inclusion of variables indicating cognition and diagnosed eye diseases and medical intervention also provided a significant improvement to the fit of the model.

**Table 4:** Likelihood ratio tests assessing contribution of sets of variables to fit of model

Variables entered in model		Pseudo *R* ^2^	LR test	(LR *χ* ^2^)	(Pr)
m0	(Null)	.000			
m1	Sex, age, ethnicity	.025	m0 nested in m1	LR *χ* ^2^(42) = 263.06	.000
m2a	Sex, age, ethnicity, wealth	.040	m1 nested in m2a	LR *χ* ^2^(35) = 151.33	.000
m2b	Sex, age, ethnicity, SSS	.042	m1 nested in m2b	LR *χ* ^2^(35) = 180.18	.000
m3	Sex, age, ethnicity, wealth, SSS	.051	m2b nested in m3	LR *χ* ^2^(35) = 94.16	.000
m4	Sex, age, ethnicity, wealth, SSS, cognitive function	.062	m3 nested in m4	LR *χ* ^2^(35) = 154.17	.000
m5	Sex, age, ethnicity, wealth, SSS, medical diagnoses and treatment	.095	m4 nested in m5	LR *χ* ^2^(35) = 454.20	.000

Results from the final model output were transformed into predicted probabilities for ease of interpretation. In this instance, these show the predicted probability of different subgroups belonging to the different trajectories identified. A positive value in Table 5 shows a respondent is more likely to belong to a particular cluster than the reference group, and a negative value shows a reduced likelihood of cluster membership compared to the reference group, all else held constant. The reference group was male, aged 60–64 years at wave 1, in the highest wealth quintile, in the highest subjective social status quintile, and had not had cataract surgery. The text reports the predicted probabilities as percentages so, for example, where the predicted probability of a female belonging to the second cluster is 0.049 in Table 5, this translates to a female being 4.9 % more likely to belong to the second cluster than the reference group. A disadvantage here is that predicted probabilities have no associated *p* value. Where significance is referred to, this relates to the original log odds model output.

**Table 5:** Predicted probabilities of cluster membership

Cluster		1	2	3	4	5	6	7	8	Mean
		Ex	Gd	*F*	*p*	G>F	E>F	G>E	*U*	
Gender										
Female		−0.055	0.049	0.005	0.000	−0.009	0.024	−0.014	0.001	0.020
Age (at wave 1)										
65–69		−0.004	0.007	−0.016	0.002	0.013	0.016	−0.017	−0.002	0.009
70–74		−0.020	−0.009	0.007	0.005	−0.003	0.027	−0.002	−0.003	0.010
75–79		−0.051	0.013	−0.007	0.005	−0.009	0.066	−0.015	−0.001	0.021
80+		−0.090	0.037	−0.006	0.005	0.004	0.090	−0.012	−0.029	0.034
Ethnicity										
Non-white		−0.147	0.111	0.062	0.008	−0.005	−0.038	0.005	0.003	0.047
Wealth										
Fourth		−0.012	0.012	0.010	0.003	−0.012	0.013	−0.016	0.003	0.010
Middle		−0.061	0.026	0.027	0.026	0.012	0.000	−0.025	−0.005	0.023
Second		−0.064	−0.032	0.047	0.016	0.026	0.004	−0.001	0.003	0.024
Lowest		−0.085	−0.058	0.082	0.016	0.036	0.018	−0.019	0.011	0.041
Missing		−0.038	−0.030	0.028	0.043	0.099	0.064	−0.113	−0.054	0.059
Subjective social status										
Fourth		−0.076	0.036	0.016	0.007	−0.003	−0.014	0.008	0.026	0.023
Middle		−0.151	0.107	0.017	0.010	0.002	−0.012	−0.014	0.041	0.044
Second		-0.154	0.065	0.029	0.016	0.012	0.002	−0.027	0.056	0.045
Lowest		−0.203	0.113	0.052	0.063	0.012	0.004	−0.081	0.040	0.071
Missing		−0.140	−0.019	0.034	0.081	0.016	−0.023	−0.044	0.095	0.056
Cognitive function										
Executive		0.049	0.031	−0.021	−0.005	−0.023	−0.021	0.007	−0.018	0.022
Memory		0.087	0.038	−0.002	−0.008	−0.022	0.000	0.010	−0.029	0.024
Treatment and eye diagnoses										
Cataract surgery		0.075	−0.076	−0.001	0.002	0.000	−0.049	0.029	0.019	0.031
Glaucoma		−0.160	0.074	0.033	0.012	0.007	0.065	−0.028	−0.004	0.048
Diabetic retinopathy		−0.120	0.064	−0.013	0.015	−0.009	0.073	0.000	−0.010	0.038
AMD		−0.185	−0.062	0.055	0.073	0.072	0.101	−0.069	0.015	0.079
Cataracts		−0.168	0.001	0.040	0.013	0.048	0.077	−0.026	0.014	0.048
Constant		0.323	0.362	0.035	0.011	0.064	0.060	0.098	0.047	

*Cluster 1* stable excellent (and slight fluctuation around excellent), *cluster 2* stable good (and slight fluctuation around good), *cluster 3* stable fair (and deterioration to fair), *cluster 4* poor and deterioration to poor, *cluster 5* gradual deterioration from good to fair, *cluster 6* rapid deterioration from excellent to fair, *cluster 7* slight improvement from good to excellent, *cluster 8* U-shaped deterioration to fair then improvement to good

Table 5 shows compared with men, women were notably more likely to be in cluster 2 and report stable good vision (4.9 %). Women were less likely to be in cluster 1 and report stable excellent vision (−5.5 %) than men. Age was a significant predictor of cluster membership. As age group at wave 1 increased, individuals were progressively less likely to be part of cluster 1; that is, compared with the youngest age group, the oldest were less likely to report stable excellent vision (−9 %). Increased age was associated with increased probability of membership of cluster 2, reporting stable good vision, cluster 4, reporting poor vision and cluster 6, reporting rapid deterioration from excellent to fair. Notable differences were seen between the predicted probabilities of cluster membership by ethnicity. Non-white respondents were less likely to report stable excellent vision (cluster 1, −14.7 %) and more likely to report stable good vision (cluster 2, 11.1 %) and fair vision and deterioration to fair vision (cluster 3, 6.2 %). However, it should be remembered the number of non-white participants in the selected sample is small (1.6 %).

The diagnosis of eye disease and the uptake of cataract surgery were significant predictors of vision trajectory. The diagnosis of an eye disease had a strong negative association with reporting stable excellent vision (cluster 1) and with reporting improvement in vision from good to excellent (cluster 7). It had a positive association with reporting fair vision and deterioration to fair (cluster 3), poor vision and deterioration to poor (cluster 4) and rapid deterioration from excellent to fair (cluster 6). Furthermore, respondents reporting age-related macular degeneration were more likely to report gradual deterioration from good to fair (cluster 5, 6.9 %) and less likely to report stable good vision (cluster 2, −6.2 %) and improvement from good to excellent vision (cluster 7, −6.9 %); respondents with diagnosed cataracts were more likely to report rapid deterioration from excellent to fair vision (cluster 6, 7.7 %) and gradual deterioration from good to fair (cluster 5, 4.8 %) and less likely to report improvement from good to excellent vision (cluster 7, −2.6 %). Having undergone cataract surgery was seen to be a significant and negatively associated with stable good vision (cluster 2, −7.6 %) and rapid deterioration from excellent to fair (cluster 6, −4.9 %) but positively associated with reporting stable excellent vision (cluster 1, 7.5 %). It must be remembered that these binary variables indicated that the eye disease was diagnosed or that cataract surgery was performed *at some point* during the 8-year observation window and the *timing* of any diagnosis or medical intervention is obscured.

Holding all else constant, social position was significantly related to cluster membership, both in terms of wealth and SSS. Decreasing levels of wealth was associated with decreased probability of more optimal vision trajectories (clusters 1 and 2) and increased probability of suboptimal trajectories (clusters 3 and 5). Compared with the highest wealth quintile, the lowest wealth quintile was 8.5 % less likely to report stable excellent vision (cluster 1) and 5.8 % less likely to report stable good vision (cluster 2), 8.2 % more likely to report fair vision and deterioration to fair vision (cluster 3), and 3.6 % more likely to report a gradual deterioration from good to fair vision (cluster 5).

Table 5 and the MNLM output confirm SSS has a significant effect on cluster membership beyond that already accounted for by wealth. Decreasing levels of SSS was associated with decreasing probability of more optimal vision trajectories (clusters 1and 7) (Table 5): compared with the highest SSS quintile, the lowest SSS quintile is 20.3 % less likely to report stable excellent vision (cluster 1) and 8.1 % less likely to report a slight improvement from good to excellent vision (cluster 7). Decreasing levels of SSS was associated increasing probability of suboptimal trajectories (clusters 3 and 4): the lowest SSS quintile compared to the highest SSS quintile was more likely to report stable fair vision and deterioration to fair vision (cluster 3, 5.2 %) and poor vision and deterioration to poor vision (cluster 4, 6.3 %).

Reporting stable good vision appears be a trajectory that is distinct to those of middle social position, both in terms of material wealth and SSS; there is an increased probability of cluster 2 for middle wealth and middle SSS which is not in keeping with the overall linear changes in level of social position.

### Cognitive function

Optimal cognitive function is associated with increased likelihood of reporting stable excellent vision (cluster 1, 4.9 and 8.7 % for executive and memory function, respectively) and stable good vision (cluster 2, 3.1 and 3.8 % for executive and memory function, respectively). It is associated with a reduced likelihood of reporting suboptimal trajectories, most notably gradual deterioration from good to fair (cluster 5, −2.3 % and −2.2 % for executive and memory function, respectively). Those reporting optimal executive function are less likely to report both stable and deterioration to fair vision (cluster 3, −2.1 %) and rapid deterioration from excellent to fair vision (cluster 6, 2.1 %) but there is no association with memory function and these vision trajectories.

### Social position, age, and trajectories of vision

**Fig. 2:**
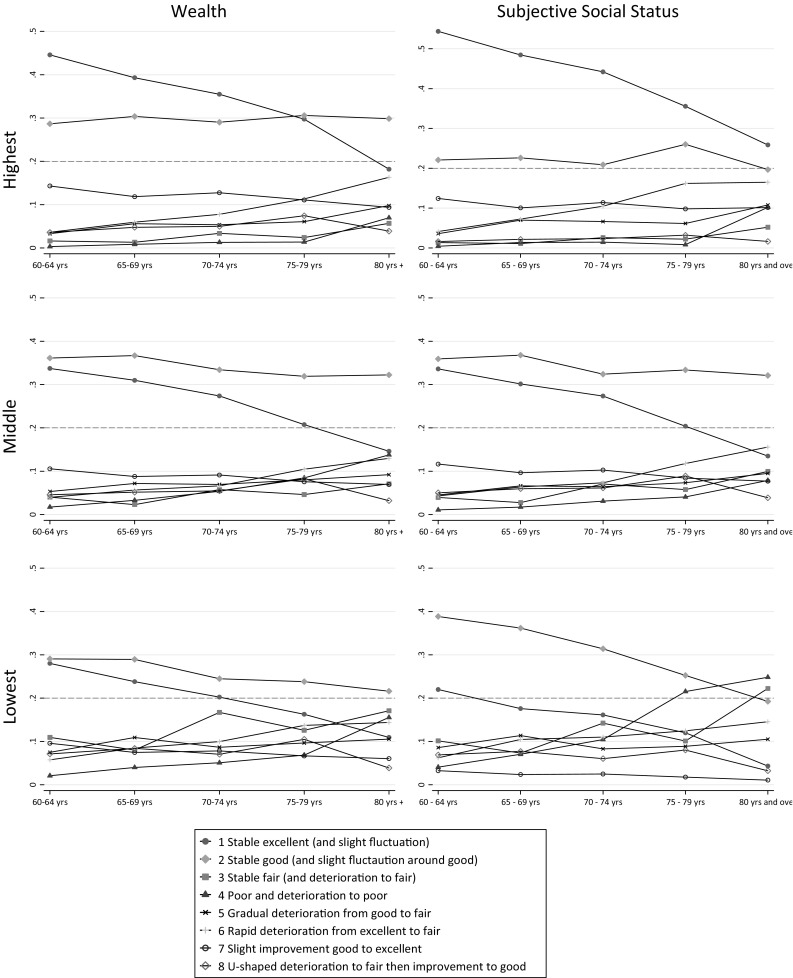
Mean predicted probability of vision trajectory by social position measures and age group

Age and both measures of social position were significant predictors of vision trajectory. Graphs were produced to show the mean predicted probabilities of cluster membership by age band at wave 1 and by wealth quintile, and separately by SSS quintile (Fig. 2, tables available upon request to the author).

For those of the highest social position, there was a clear separation between the probabilities of reporting stable optimal vision trajectories (clusters 1 and 2) and the remaining trajectories. Controlling for the effects of other variables, the highest quintiles were likely to report stable excellent vision or stable good vision more than any other trajectory irrespective of age; the probability of being part of cluster 2 did not drop down below 0.2, even in the oldest cohort, and the probability of being part of cluster 1 only dropped below 0.2 for the highest wealth quintile at the very oldest age (mean predicted probability .190). In the highest *wealth* quintile, the probability of reporting stable excellent vision decreased with an increase in age group while the probability of reporting stable good vision marginally increased with age; only for those aged 75–79 and 80+ at wave 1 was the probability of reporting stable excellent vision less than that of reporting stable good vision. Similarly, the highest SSS quintile was more likely to report stable excellent vision or stable good vision than any other trajectory. However, among those ranking themselves as being of high social position, the probability of reporting stable excellent vision was the most highly reported trajectory across *all* age bands. The mean predicted probability of reporting a suboptimal vision trajectory (clusters 3–8) was low among the youngest group in the highest wealth quintile, all with predicted probabilities between 0.004 and 0.039. This is also seen in those in the highest SSS quintile. In both measures of social position, the probability of reporting a suboptimal vision trajectory increased with the increase in age band. It must be noted, though, that reporting stable fair vision or poor vision (cluster 3 or 4) remained among the least probable trajectories in the highest wealth and highest SSS quintiles, even among the oldest ages.

For those of middle social position, the three most probable vision trajectories in the youngest age band were stable excellent vision, stable good vision, and slight improvement from good to excellent vision (clusters 1, 2, and 7). On both measures of social position, with the increase in age band there was an increased dispersion in the mean predicted probabilities of reporting different trajectories. Reporting stable good vision (cluster 2) was the most likely reported vision trajectory of the middle quintiles even among those in the youngest age bands (unlike the highest quintiles, where excellent vision tended to be the most probable trajectory). The probabilities of reporting both stable excellent and stable good vision (clusters 1 and 2) declined with each age band: stable good, only marginally so, and stable excellent, more dramatically. For those in the middle wealth quintiles, there was a notable difference in the probability of reporting poor vision (cluster 4) between the youngest and oldest age bands, from 0.018 to 0.144. This increase meant that for the youngest age band in the middle wealth quintile, this was the least reported trajectory yet the third most commonly reported trajectory for the oldest age band. For those in the middle SSS quintile, there was a notable difference in the probability of reporting deterioration from excellent to fair vision (cluster 6) between the youngest and oldest age bands, from 0.042 to 0.153. Membership of cluster 6 was the second least probable trajectory to be reported by the youngest category but the second most likely trajectory among the oldest age band.

In the lowest wealth quintile there was a clear separation between the mean predicted probabilities of reporting stable optimal vision trajectories (clusters 1 and 2) and the remaining trajectories by the youngest category; with the increase in age band there was a convergence of the mean predicted probabilities across the trajectories, with no such separation, unlike the highest and middle wealth quintiles. For the oldest group in the lowest wealth quintile, only stable good vision was likely to be reported by more than 20 % of people (cluster 2, 0.218); while stable excellent vision, fair vision, poor vision, and both deterioration trajectories were likely to be reported by 10–20 % (clusters 1 and 3–6) and less than 10 % of this group were likely to report an improvement in vision (cluster 7, 0.058), or a u-shaped deterioration followed by improvement (cluster 8, 0.038). The youngest age group in the lowest SSS quintile reported stable good vision (cluster 2, 0.385) as opposed to stable excellent vision (cluster 1, 0.221), which is distinctive from the youngest in lowest wealth quintile that are equally likely to report stable excellent and stable good vision trajectories (0.277 and 0.291, respectively). Among the oldest age group in the lowest SSS quintile, poor vision and deterioration to poor vision (cluster 4) and fair vision and deterioration to fair vision (cluster 3) were the most probable trajectories reported (0.233 and 0.225, respectively), even more likely than stable good vision (cluster 2, 0.205).

## Discussion

Optimal matching, hierarchical clustering, and multinomial logistic regression were used to describe the sequential data, produce a typology of vision trajectories, and examine the socio-demographic characteristics associated with different trajectories. An eight category typology was found to provide a suitable representation of the trajectories of vision from the sample of 2956 respondents, aged 60 and over, who self-classified their vision in 5 waves of ELSA over an 8-year period. Clusters 1–3 depict trajectories of stable vision (stable excellent, good, and fair), cluster 4–6 represent deterioration in vision (poor and deterioration to poor vision, gradual deterioration, and rapid deterioration), and clusters 7 and 8 show an improvement in vision (slight improvement and u-shaped deterioration followed by improvement). While longitudinal weights were used to adjust for survey non-response, findings are potentially biased towards those who are healthy enough participate across all five waves, which perhaps underestimates the proportion of people with less than optimal vision trajectories and the relationship between social position and vision change. Findings are also biased towards those who are younger, with better vision and lower incidences of eye diseases.

In this study, onset of diagnosed eye diseases was associated with trajectories of rapid deterioration in vision, fair vision, and poor vision. Timely diagnosis and treatment of eye diseases can substantially reduce the incidence and prevalence of vision loss in older people. As a number of eye diseases are detectable before symptoms present themselves to the individual, regular eye tests for those aged 60 years and over may reduce preventable and treatable vision loss and therefore the magnitude of association between onset of diagnosed eye disease and suboptimal vision trajectories. Currently, between the ages of 60–69, people are entitled to a free eye test every 2 years; increasing the frequency of this entitlement on the NHS and paying attention to barriers to the take up of this entitlement may lead to increased rates of early detection and treatment of the most common age-related eye diseases.

The analysis shows marked differences across quintiles of social position, highlighting the magnitude of health inequalities experienced by older people as they age. Clusters 3, 4, 5, and 6 represent over a fifth of the study population and show stable suboptimal vision or deterioration in vision, and there is a particular importance in focusing on the poorer social characteristics of the individuals in these clusters if measures to prevent or improve vision loss are to succeed. As well as social position being related to vision per se, these findings may also indicate social inequalities have an effect on the identification and treatment of refractive errors and eye disease and therefore on visual function. A recent ELSA report suggested access to healthcare services is limited among those with lower wealth, with 15.6 % of men and women in the lowest wealth quintile stating they find it difficult to access an optician when needed compared with 4.2 % of men and 3.7 % of women in the highest wealth quintile (Matthews and Nazroo 2014). Relatedly, people in lower social positions may have undiagnosed eye conditions, in turn impacting their self-reported vision. Existing research has shown that for older people the cost of glasses presents a barrier to having eye tests, particularly for those in lower income brackets (Conway and McLaughlan 2007). There is a potential for raising awareness and uptake of entitlement to NHS optical vouchers among lower income groups (Conway and McLaughlan 2007), increasing voucher amounts, and increasing the availability and choice of low cost glasses that can be covered by the voucher amount (Jessa et al. 2009). Removing barriers to regular eye examinations would increase the likelihood of early identification and treatment of refractive errors and eye disease in the lowest quintiles. These changes have the potential to impact the self-reported visual function of those who are most at risk of suboptimal vision trajectories. Alongside an emphasis on the role of healthcare provision, and given that the costs of eye exams are covered under the NHS, these findings might also indicate an area for further research into how to encourage self-management of eye care among those in lower socioeconomic positions.

The results of the study may be limited in that the only medical procedure pertaining to vision deterioration included ELSA is cataract surgery. The significant association of this variable to trajectories of vision indicates other, unrecorded, medical procedures are also likely to have significantly impacted on the observed trajectories. Anti-VEGF treatments are currently used for improving vision among patients with age-related macular degeneration, diabetic maculopathy and macular oedema, all of which are conditions likely to be affecting vision trajectories in the ELSA population.

The use of a self-reported measure of vision is perhaps less than ideal as responses reflect more than objective visual acuity. However, objective visual acuity may be insufficient for detecting visual impairment in older people; many people with good acuity are effectively visually impaired in performing everyday tasks under non-ideal conditions involving low and changing light levels, glare, and low contrast (Brabyn et al. 2001; Haegerstrom-Portnoy et al. 1999). Therefore, a self-report measure, reflecting vision under the non-optimal viewing conditions of daily life, may be a more accurate assessment of vision. Whillans and Nazroo (2014) compared self-reported vision with objective acuity (logMAR) and found subjective vision was a significant predictor of objective low acuity in older people (97 % of those self-reporting normal vision measured normal objectively). The study also found those in lower social positions were more likely to incorrectly self-report visual impairment than wealthier individuals.

The findings in this study may have limited applicability to the US given the notable differences between healthcare systems in England and the US: In England, routine eye exams to the over 60 s and medical treatment are publically funded, and service provision is not related to an ability to pay at the point-of-delivery. In the US, healthcare is funded by a combination of public and private insurance. Generally speaking, Medicare does *not* cover routine vision services like eye exams and glasses; it only covers eye care services if a chronic eye condition is suspected or has been diagnosed, glaucoma screening for those considered high risk, and surgical procedures (e.g., cataract surgery); however, recipients are required to pay a coinsurance, creating a significant point-of-service fee for many users. Given the patchwork of public and private insurance in the US and the coverage offered by Medicare, those least able to afford comprehensive health insurance or point-of-service fees may be more likely to live with uncorrected refractive errors and undiagnosed (yet detectable and treatable) eye conditions, effecting vision. Thus, if the *potential* financial cost of glasses constitutes a significant barrier to the uptake of a free eye exam and individuals’ self-management of eye care in England, manifesting itself in systematic and empirically evidenced social inequalities in vision, this may suggest the relationship between social position and vision trajectories is even stronger in the US. It should also be noted that the small number of non-white participants in the ELSA sample (1.6 %) may present issues with the external validity of this study, especially when generalising findings to US populations. The findings from this study invite further research into the effects of social inequalities, and it is interrelationship with healthcare provision, on the eye health and patterns of vision change of older people in the US.
